# Electric Fields and Charge Separation for Solid Oxide Fuel Cell Electrodes

**DOI:** 10.1021/acs.nanolett.2c02468

**Published:** 2022-09-06

**Authors:** Nicholas J. Williams, Ieuan D. Seymour, Dimitrios Fraggedakis, Stephen J. Skinner

**Affiliations:** † Department of Materials, Imperial College London, Exhibition Road, London SW7 2AZ, U.K.; ‡ Department of Chemical Engineering, Massachusetts Institute of Technology, Cambridge, Massachusetts 02139, United States; § Department of Chemical and Biomolecular Engineering, University of California, Berkeley, California 94720, United States

**Keywords:** DFT, SOFC, electric field, surface potential, thermodynamics

## Abstract

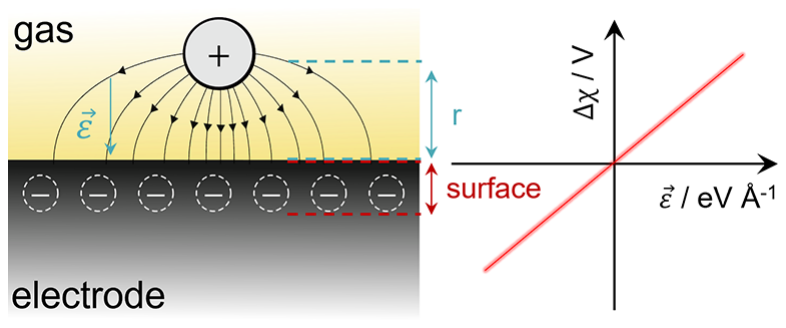

Activation losses at solid oxide fuel cell (SOFC) electrodes have been widely attributed to charge transfer at the electrode surface. The electrostatic nature of electrode–gas interactions allows us to study these phenomena by simulating an electric field across the electrode–gas interface, where we are able to describe the activation overpotential using density functional theory (DFT). The electrostatic responses to the electric field are used to approximate the behavior of an electrode under electrical bias and have found a correlation with experimental data for three different reduction reactions at mixed ionic–electronic conducting (MIEC) electrode surfaces (H_2_O and CO_2_ on CeO_2_; O_2_ on LaFeO_3_). In this work, we demonstrate the importance of decoupled ion–electron transfer and charged adsorbates on the performance of electrodes under nonequilibrium conditions. Finally, our findings on MIEC–gas interactions have potential implications in the fields of energy storage and catalysis.

Electrochemical devices, such as solid-oxide fuel cells (SOFCs), allow for reversible chemical to electrical energy conversion, with efficiency surpassing that of the combustion engine.^1^ The Faradaic reactions at the fuel (i.e., H_2(g)_ or CO_(g)_) and air electrodes of an SOFC can be given simply as^2,3^
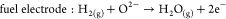
1
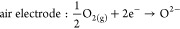
2where O^2–^ and e^–^ represent oxide ions in the electrolyte and electrons in the electrode, respectively. The dipole at the electrode–liquid electrolyte interface, known as the activation overpotential (η_act_), accounts for the energy barrier of charge-transfer processes such as Li-ion intercalation.^4−8^ For SOFC systems, these phenomena do not apply, and little is known about the electrode–gas interactions that determine the activation overpotential. The ambipolar transfer of ions and electrons with the gas phase means that this interface is chemical in nature.^9^ However, in the limit where one of these charge-transfer processes is particularly slow, the adsorbed gas will become charged and will therefore impose an electrostatic surface potential (χ) across the electrode–gas interface.

The process of gas reduction at a MIEC surface can be generalized as^10,11^
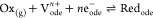
3where Ox_(g)_, V_ode_^*n*+^, e_ode_^–^ and Red_ode_ represent the oxidized gas species (i.e., O_2(g)_ or H_2_O_(g)_), the vacant surface site, mobile electrons, and the reduced species, respectively. Superscript *n* represents the charge of the vacant site and the number of electrons consumed by an ambipolar charge-transfer reaction. In the theory outlined by Fraggedakis et al., Faradaic reactions at an electrode–electrolyte interface can proceed through a coupled ion–electron transfer (CIET) mechanism.^4^ The excess chemical potential landscape in Figure 1a–c illustrates three scenarios of ion–electron transfer. When the barrier for ion transfer (IT) is significantly lower than that for the possible electron-transfer (ET) step (Figure 1a), the charge-transfer process is decoupled. As such, the adsorbate will hold the charge of the vacancy which it filled and will develope an electrostatic surface potential. In the second case (Figure 1b), both IT and ET can occur simultaneously through a concerted mechanism. In such a situation, there is no stable intermediate state and no charged adsorbate exists on the electrode surface. Therefore, charge separation does not occur, and no electrostatic surface potential is observed. Last, when the energy barrier for IT is significantly larger than ET, the gas species will be reduced before the rate-limiting IT step (Figure 1c). Similar to the ET-limited case, the charge-transfer process is decoupled and the adsorbate will hold a charge equal to the number of electrons transferred in the ET step, resulting in an electrostatic surface potential.

**Figure 1:**
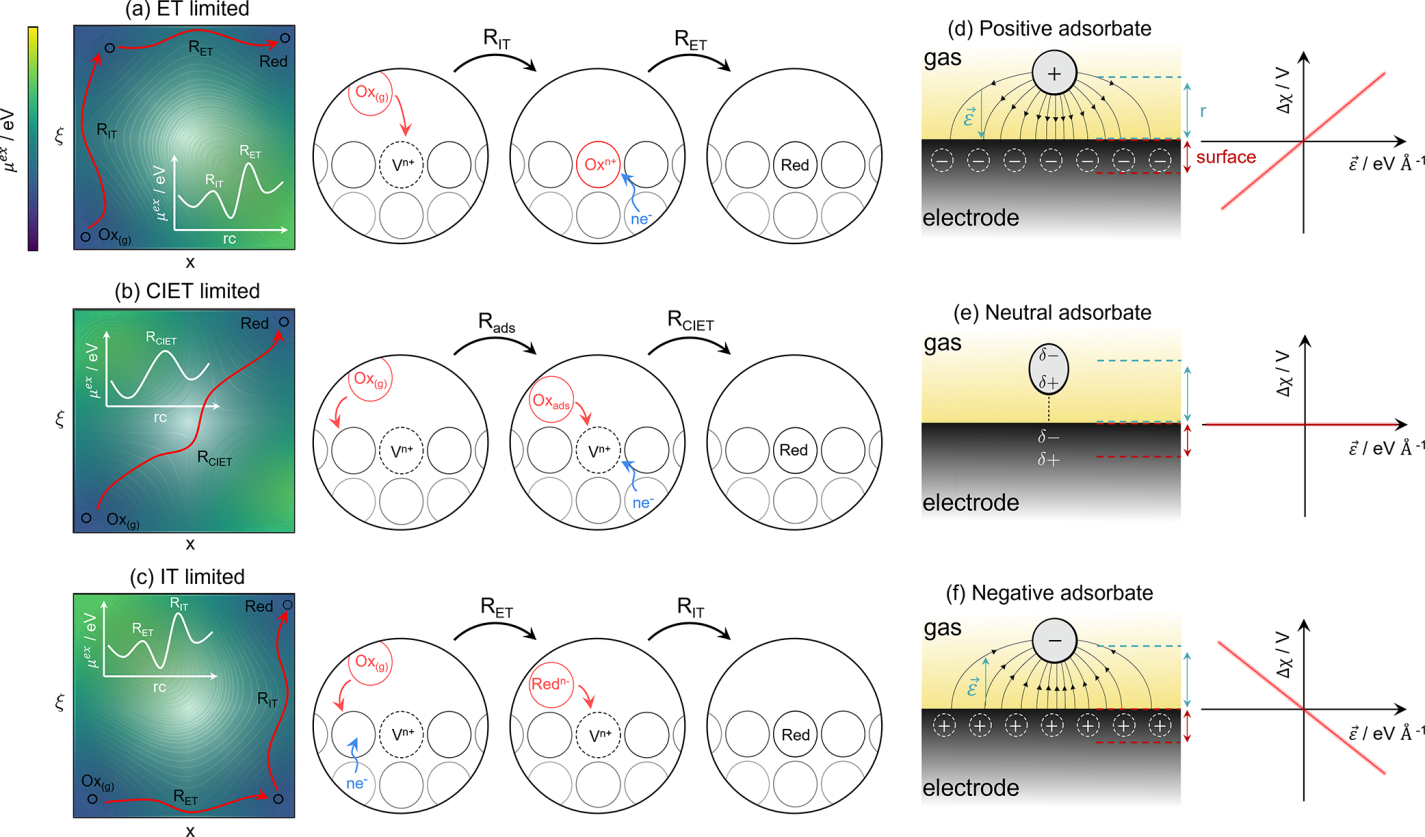
Left-hand column details the excess chemical potential energy landscape for (a) ET limited reduction, (b) CIET limited reduction, and (c) IT limited reduction. The planes represent the ET reaction coordinates (*x*) and the IT reaction coordinate (ξ).^4^ The white plots represent the 1D chemical potential landscape explored by the reduction reaction. *R*_ads_ is the adsorption step, *R*_ET_ is the electron-transfer (ET) step, *R*_IT_ is the IT step, and *R*_CIET_ is the CIET step. The right-hand column details a schematic illustration of the shift in electric potential of an electrode experiencing a negative overpotential, where the black and red lines represent the potential at the point of zero charge (PZC) and under a negative applied electric field, respectively. (d) Positive adsorbate induces an electric field vector pointing into the electrode surface, where Δχ – η_act_ has a positive correlation, (e) a neutral adsorbate induces no electric field with no correlation, and (f) a negative adsorbate induces an electric field vector pointing away from the electrode surface, yielding a negative Δχ – η_act_ correlation.

In a previous study, we determined that the electrostatic surface potential had a profound influence on the gas reduction kinetics, where a Δχ ≠ 0 relationship is desirable.^12,13^ Few experiments have investigated the complex Δχ – η_act_ relationship, leaving this phenomenon poorly understood despite its kinetic merit.^12,14−17^ By analyzing the shift in the outer work function using *operando* X-ray photoelectron spectroscopy (XPS) over an applied overpotential range, Feng et al. measured the Δχ – η_act_ relationship for three different electroreduction systems illustrated in Figure 2. Mechanistic details given in Table 1 and Table S1 show the overpotential derived from each reaction. Upon expanding the electrochemical potential terms, we find the general solution *neη*_act_ = ±*T*Δ*S* ± *ne*Δχ. Thus, each derivation finds a relationship consistent with which electrons and adsorbates interact.^9,18^ They also concluded that the space–charge potential at the electrode surface was invariant under an applied overpotential (i.e., H_2_O and CO_2_ on CeO_2_; O_2_ on LaFeO_3_).^9^ While this finding points toward the existence of an active mechanism controlling the electrochemical performance of electrified interfaces, the theoretical understanding is still in its early stages. Here, we rationalize the mechanistic basis of these observations.

**Figure 2:**
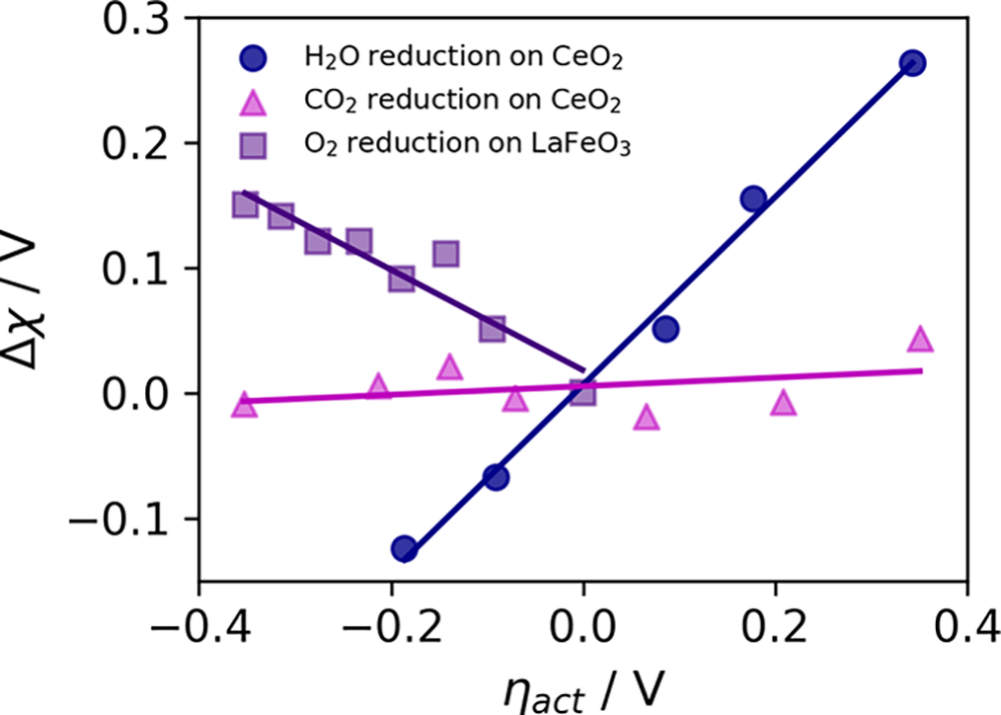
Experimental shift in the electrostatic surface potential as a function of the overpotential relationship at the Sm_0.2_Ce_0.8_O_1.9_–gas interface in 0.35 mbar 1:8:4 H_2_/H_2_O/Ar (blue circles), 0.36 mbar 2:25 CO/CO_2_ (magenta triangles), and La_0.8_Sr_0.2_FeO_3_ in 1.3 mbar O_2_ (purple squares).^9,18^ The solid line represents the linear fit to the experimental data with gradients ∂Δχ/*∂η*_act_ = 0.75, 0.03, and −0.40 for H_2_O reduction, CO_2_ reduction, and O_2_ reduction, respectively.

**Table 1:** Steps in Gas Reduction at the MIEC Surface for Three Systems Investigated Experimentally, Written in Kröger–Vink Notation, Where the Reported Rate-Limiting Step for Each Process Is Given in Bold^9,15,19−21^^a^

H_2_O reduction on CeO_2_	
H_2_O_(g)_ + V_O_^••^ + O_O_^*x*^ ⇌ 2OH_O_^•^	2*e*η_act_ = −*T*Δ*S*_conf_ – 2*e*Δχ
**2OH_O_^•^** **+ 2Ce**_**Ce**_^′^ **⇌** **H**_2(**g**)_ **+ 2O**_**O**_^***x***^ **+ 2Ce**_**Ce**_^***x***^	2*eη*_act_ = −*T*Δ*S*_conf_ + 2*e*Δχ

CO_2_ reduction on CeO_2_	
CO_2(g)_ + O_O_^*x*^ ⇌ CO_3__O_^*x*^	2*eη*_act_ = −*T*Δ*S*_conf_
**CO**_3_**O**__^***x***^ **+ 2Ce**_**Ce**_^′^ **+** **V_O_^••^** **⇌** **CO**_(**g**)_ **+ 2O**_**O**_^***x***^ **+ 2Ce**_**Ce**_^***x***^	2*eη*_act_ = −*T*Δ*S*_conf_

O_2_ reduction on LaFeO_3_	
^1^/_2_O_2(g)_ + Fe_Fe_^*x*^ ⇌ O_ads_^′^ + Fe_Fe_^•^	*eη*_act_ = *T*Δ*S*_conf_ + *e*Δχ
O_ads_^′^ + V_O_^••^ + Fe_Fe_^*x*^ ⇌ O_O_^*x*^ + Fe_Fe_^•^	*eη*_act_ = *T*Δ*S*_conf_ – *e*Δχ

a For H_2_O reduction, steam adsorption is an IT step which consumes an oxygen vacancy (V_O_^••^) and forms two hydroxyls (OH_O_^•^) on the surface. This is followed by the rate-limiting ET step which consumes two polarons (Ce_Ce_^′^) and forms H_2(g)_. For CO_2_ reduction, the first step forms an adsorbed neutral carbonate  which then undergoes the rate-limiting CIET step to form CO_(g)_. For O_2_ reduction, we assume fast dissociate adsorption and ET forming a negatively charged adsorbate (O_ads_^′^) and a polaronic hole (Fe_Ḟe_). The second step is the rate-determining incorporation step which includes a single electron transfer and consumes an oxygen vacancy.^15^ The activation overpotential equations are given for each reaction step derived in Table S1, where Δ*S*_conf_ is the change in configurational entropy under nonequilibrium.

As defined by Bazant, the internal energy, or open circuit voltage (*V*_0_), of a uniform reactive mixture is given as the first variational derivative of the Gibbs free energy (*G*) with respect to the concentration^8^
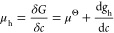
4where μ^Θ^ and *g*_h_ represents the standard chemical potential and homogeneous free energy density of the mixture, respectively. The reservoir chemical potential (μ_res_), or cell voltage (*V*), acts as the nonequilibrium chemical potential of the system.^22^ The difference between the internally controlled potential (μ_h_) and the externally controlled potential (μ_res_) is given by the reaction affinity, *A* = μ_res_ – μ_h_, which controls the rate of a reduction reaction.^22^ This can also be expressed as the activation overpotential^4,7,8^
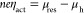
5where *n* is the number of electrons transferred in the Faradaic reaction. The Fermi energy (*E*_F_) describes the electrochemical potential of free electrons, and the shift in the Fermi energy describes the activation overpotential at each electrode (illustrated in Figure S1)^9,19^
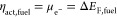
6
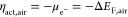
7where under a positive overpotential (fuel cell mode) gas is oxidized by the fuel electrode and reduced by the air electrode (full derivation in SI). The formation of the electrostatic surface potential can be described as the difference in electrostatic potential of free electrons in the electrode (ϕ_e_) and the adsorbate (ϕ_ad_):^12,16^
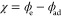
8

Under bias, an electrostatic potential shift is defined at the surface as
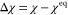
9where an effective electrical double layer is formed between the electrode surface and the adsorbed species.

The interface between the adsorbate and the electrode can be understood as a parallel plate capacitor where the electric field (E⃗) is controlled by the voltage (*V*) and the distance (*r*) between the adsorbed species and the first layer of the surface, E⃗ = *V*/*r*. However, modulating the adsorbate–electrode distance will result in an energetically unfavorable distortion of the bonding. Therefore the shift in the electrostatic surface potential is a result of a change in the coverage of polar adsorbates such that  where , ε_0_, ρ_0_, and θ represent the dipole moment normal to the surface, vacuum permittivity, density of available adsorption sites, and adsorbate coverage, respectively.^16^ A positively charged adsorbate (Figure 1d) imposes a negative electric field vector which points into the electrode surface . A neutral adsorbate (Figure 1e) has no electrostatic attraction to the electrode and is therefore weakly bound to the oxide surface by dipolar interactions. Finally, a negatively charged adsorbate (Figure 1f) imposes a positive electric field which points away from the electrode surface.

Advances in the modeling of electrochemical interfaces have allowed computational chemists to study charged surfaces by incorporating electric fields into electronic structure simulations using density functional theory (DFT) as illustrated in Figure S3.^5,23^ For electrochemical processes which induce the polarization of electron density, the corresponding reaction energy depends on the electrochemical potential of the surface, where the effects of the electric field are found to be strongly dependent on its dipole moment and polarizability, according to^5,23,24^
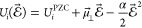
10where *U*_*i*_^PZC^ is the internal energy at the point of zero charge and α is the polarizability. Equation 10 implies that a surface with a positive dipole moment is stabilized by a negative electric field vector and vice versa. As such, the electrostatic surface potential can be described by the difference in dipole moment between the products and reactants, ,
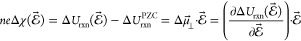
11where Δ*U*_rxn_ = *U*_p_ – *U*_r_ is the change in internal energy of the reduction reaction. The electrostatic potential experienced by mobile charges in the MIEC electrode phase will shift in accordance with the activation overpotential, as described by eqs 6 and 7.^25^ This is analogous to applying an electric field, where the Fermi energy response can be used to determine the activation overpotential:^19,24^
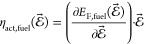
12
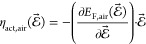
13

It is therefore possible to predict the origin of the activation overpotential for adsorbed gas species by combining eq 11 with eq 12 or 13.^5^ In this work, using a novel computational framework, we are able to predict the η_act_ – Δχ relationship of an electrode–adsorbate system using applied electric fields.

DFT calculations were carried out to determine the internal energy (*U*) of the reactant and product configurations of the rate-limiting step for the reduction reactions detailed in Table 1 as a function of the applied electric fields (Figure 3a–c). By taking the energy difference between the reactants and products, the reduction driving force (eq 11) is given as a function of electric field (Figure 3d–f). We then determined the shift in Fermi energy of the surface with free charges (Ce_Ce_^′^ or Fe_Fe_^•^) as a function of the appled electric field (Figure 3g–i). The simulated Δχ – η_act_ relationship for the three systems (Figure 3j–l) considered is given in Table S2.

**Figure 3:**
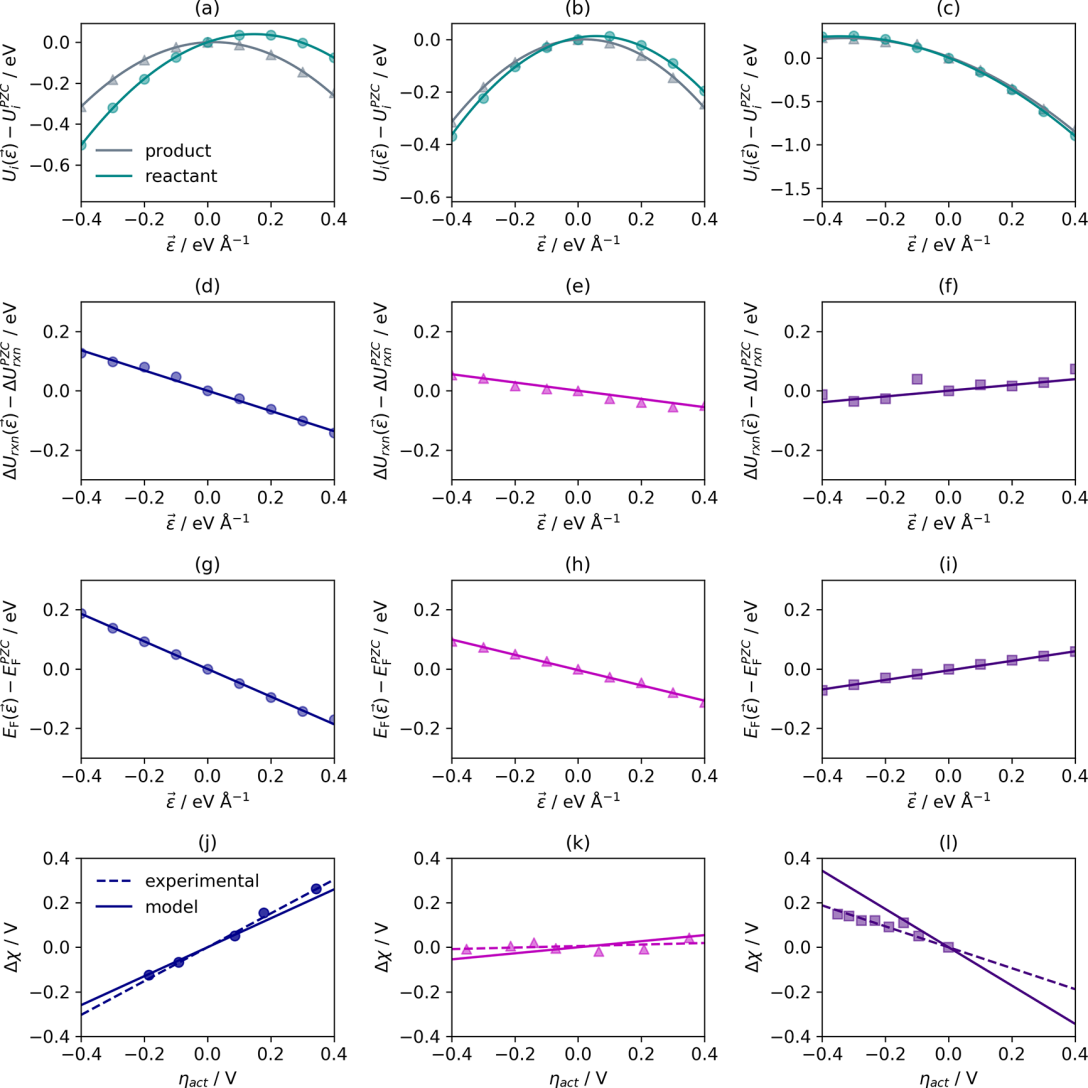
(a–c) Internal energy calculation as a function of the applied electric field fit to eq 10, (d–f) internal energy of the reaction response to an applied electric field where solid lines represent the fit to eq 11, (g–i) Fermi energy response to an applied electric field, where solid line represents the fit to eq 12 and 13, and experimental shift in the electrostatic surface potential as a function of the overpotential relationship at the Sm_0.2_Ce_0.8_O_1.9_–gas interface in (j) 0.35 mbar 1:8:4 H_2_/H_2_O/Ar (blue circles),^9^ (k) 0.36 mbar 2:25 CO/CO_2_ (magenta triangles),^9^ and (l) La_0.8_Sr_0.2_FeO_3_ in 1.3 mbar O_2_ (purple squares).^18^ The dashed and solid lines represent the linear fit to the experimental data and simulated results, respectively.

For H_2_O reduction on CeO_2_, the gradient  is in good agreement with the linear fit to the experimental data ∂Δχ/*∂η*_act_ = 0.76 (Figure 3j). The error can be attributed to lateral interactions between dipoles which occur when the adsorbate coverage is greater than approximately 1%, where the intrinsic dipole moment imposed by the adsorbate is dependent on the coverage.^12,16^ DFT calculations were carried out at , which is close to the experimentally measured coverage at PZC and is where the model and data best agree. However, as η_act_ is increased, the model and experimental results deviate as the hydroxyl coverage decreases, increasing the strength of the intrinsic dipole moment and increasing the gradient ∂Δχ/*∂η*_act_. Mechanistically, the ambipolar charge-transfer reaction is decoupled, where the fast adsorption step (Table 1) fills a charged oxygen vacancy (IT) and the ET step becomes rate-limiting. Table 1 illustrates the Δχ – η_act_ relationships derived from each reaction step, where we have shown that ET is rate-limiting to derive the experimental trend Δχ ≈ η_act_.

For CO_2_ reduction on CeO_2_, the gradient  also agrees with the linear fit to the experimental data ∂Δχ/*∂η*_act_ = 0.03 (Figure 3k). The true CeO_2_ surface will have many arrangements of the electronic defects, causing the net electrostatic potential to cancel. Here we have analyzed only one such defect complex which has a relatively small dipole moment , accounting for the model’s overestimation of the electrostatic surface potential. With respect to the surface chemistry, the ambipolar charge-transfer reaction remains coupled. As such, there is no stable charged adsorbate state, so a negligible electrostatic surface potential is observed.

Contrary to previous suggestions regarding the magnitude of the electrostatic surface potential, fast kinetics are not entirely based upon the strength of the intrinsic dipole moment of the adsorbate.^15,16^ As we observed for CO_2_ reduction on CeO_2_, the intrinsic dipole moment of the carbonate is relatively large , yet the shift in electrostatic surface potential is zero. This results from the neutrality of the adsorbate  and the absence of charge separation, meaning that the carbonate will experience a change in the chemical potential only under an applied overpotential (Table 1).^9^

For O_2_ reduction on LaFeO_3_, the gradient  while the linear fit to the experimental data ∂Δχ/*∂η*_act_ = −0.47 (Figure 3l).^18^ The general trend is captured correctly; however, the model overestimates the shift in the electrostatic surface potential. The most obvious reason for the error observed in Figure 3l is the simplicity of the model, where strontium was not included in the DFT calculation. We chose to use Fe on the surface layer because it was reported to be the most stable termination under operational conditions.^26^ However, we found that using strontium or lanthanum in the terminating layer had a negligible effect on the dipole moment of the surface. Additionally, doping strontium into the subsurface layer also had a negligible effect on the dipole moment of the surface. Guan found inconsistencies in the ∂Δχ/*∂η*_act_ relationship with respect to variations in the La/Sr ratios.^18^ Guan also observed that the ∂Δχ/*∂η*_act_ relationship was dependent on the  value of the environment. This suggests that the shift in surface potential was limited by the equilibrium potential. Fleig proposed that the buffering effect was due to the limited coverage of adsorbed gas on the surface at the PZC.^27^

With respect to the mechanism for charge transfer, the ambipolar charge-transfer reaction has been decoupled by slow IT. This agrees with the derivation in Table 1, where the IT step yields the experimentally given relationship Δχ ≈ −η_act_. While we strongly believe that the results of this study show that O_2_ reduction on MIEC surfaces is driven by Δχ, we must also note that the exact mechanism is subject to discord. Therefore, we do not intend to speculate further on the mechanistic details in this study.

We have described the effect of electric fields on the electrostatic surface potential at the MIEC–gas interface. By integrating an electric field with first-principles calculations, we validated the model to correctly predict the nature of the electrostatic surface potential for three experimentally studied systems. Furthermore, we have determined a link between the electrostatic surface potential and the mechanistic nature of the rate-limiting charge-transfer reaction, where we illustrated the importance of decoupled charge transfer for optimum kinetics. These kinetic effects may also be applied to other MIEC systems for energy storage and conversion.

## Calculation Methods

Spin-polarized density functional theory (DFT) calculations were carried out using the Vienna *Ab initio* Simulation (VASP) code.^28^ The ionic cores were described by PAW potentials (an O pseudopotential was used for oxygen), and the wave functions were expanded in plane waves with an energy cutoff at 520 eV.^29^ The PBE-generalized gradient approximation (GGA) was used.^30,31^ To describe the Ce 4f and Fe 3d electrons, DFT+U was implemented using the Dudarev treatment.^31−33^ For Ce 4f electrons, we used *U*_eff_ = 5 eV following the work of Castleton et al., and for the Fe 3d electrons, we used *U*_eff_ = 3 eV following the work of Grau-Crespo et al.^34,35^ The surfaces were modeled as symmetric slabs with a thickness of 12 atomic layers and 3 × 3 cell expansion in the lateral directions. The bottom three atomic layers were fixed during geometry optimizations. The periodic images of the slab were separated along the *c* direction by a vacuum region of about 15 Å. The convergence parameters for electronic and ionic relaxation were set to 10^–7^ and 10^–4^ eV, respectively, to guarantee the sufficient accuracy of the calculated forces. The dipole correction was used to decouple the electrostatic interaction between the periodic images. The calculations were performed with a 4 × 4 × 1 Monkhorst Pack grid. For gases, electronic calculations were carried out in a 13 × 14 × 15 Å^3^ box. The standard chemical potential was calculated as μ^o^ = *E*^el^ + ZPE – *TS*, where the entropy of the gas and surface was calculated using the ASE thermochemistry package.^36^ Electric fields were implemented using the EFIELD tag.^37^

### H_2_O Reduction on CeO_2_

The CeO_2_(111) termination was studied because it was previously reported to be the most stable termination under solid oxide cell operational conditions.^38^ Additionally, Feng et al. speculated that the SDC thin film grown on the current collector was (111)-oriented.^39^ The rate-limiting step of H_2_O reduction on CeO_2_ was reported to be ET (Table 1), where we calculate the free energy of the pristine slab and a slab with a singly charged OH_O_^•^ adsorbate (Figure S2a,b).^20^

### CO_2_ Reduction on CeO_2_

Studies on the CeO_2_(110) termination reported that the singly charged CO_3_O__^•^ state was stable during the reduction of CO_2_.^21,40^ However, no stable intermediate states were found when exploring the reduction of CO_2_ on the CeO_2_(111) termination. The rate-limiting step was determined to be CIET, where we calculated the free energy of the pristine slab and a neutral  state on a reduced CeO_2_(111) slab (Figure S2c,d).

### O_2_ Reduction on LaFeO_3_

The LaFeO_3_(100) termination with Fe on the surface layer was reported to be the most stable termination under operational conditions.^26^ The IT was reported to be the rate-limiting step (Table 1),^15^ where we calculated the free energy of the pristine slab and a charged O_O_^•^ state in which the polaronic hole was located at Fe_Fe_^•^ (Figure S2e,f).

## Supporting Information Available

Schematic of the potential across the fuel cell at open circuit voltage (*V*_cell_), in fuel-cell mode (*V*_FC_), and in electrolysis mode (*V*_EC_); schematic of the adsorbate state for 2OH_O_^•^ on CeO_2_,  on CeO_2_, and O_O_^′^ on LaFeO_3_; derivations of the activation overpotential; schematic illustration of the potential with no applied field and an applied field; Fermi energy, internal energy, and electrostatic surface potential of the reaction as a function of the electric field vector; and derivation of eqs 6 and 7 (PDF)
