# Supplementary material for: Electric Fields and Charge Separation for Solid Oxide Fuel Cell Electrodes
Source: Nano Lett. 2022 Sep 6;22(18):7515–21. doi: 10.1021/acs.nanolett.2c02468 (PMC9523703; doi:10.1021/acs.nanolett.2c02468)
Supplement: Supplementary file 1 — nl2c02468_si_001.pdf [file nl2c02468_si_001.pdf]

# Electric Fields and Charge Separation for Solid Oxide Fuel Cell Electrodes

Nicholas J. Williams,*,†,‡ Ieuan D. Seymour,† Dimitrios Fraggedakis,¶ and Stephen J. Skinner†

†*Department of Materials, Imperial College London, Exhibition Road, London SW7 2AZ, UK*

‡*Department of Chemical Engineering, Massachusetts Institute of Technology, Cambridge, MA, 02139, USA*

¶*Department of Chemical and Biomolecular Engineering, University of California, Berkeley, CA-94720, USA*

E-mail: nw7g140@gmail.com

## Supplementary Information

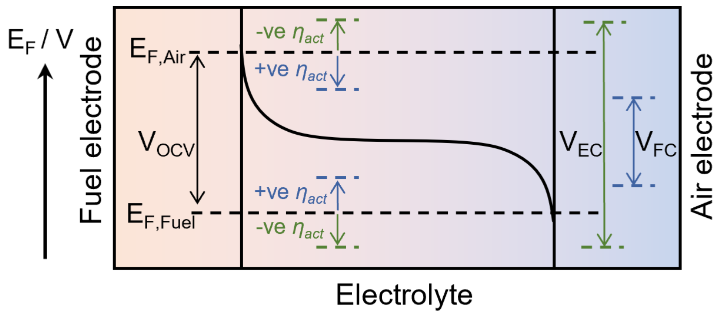


Figure S1: Schematic of the potential across the fuel cell at open circuit voltage ($V_{Cell}$), fuel cell mode ($V_{FC}$) and electrolysis mode ($V_{EC}$). The Fermi energy ($E_{\mathrm{F}}$) of fuel and air electrodes is also given as a function of the electric field vector.

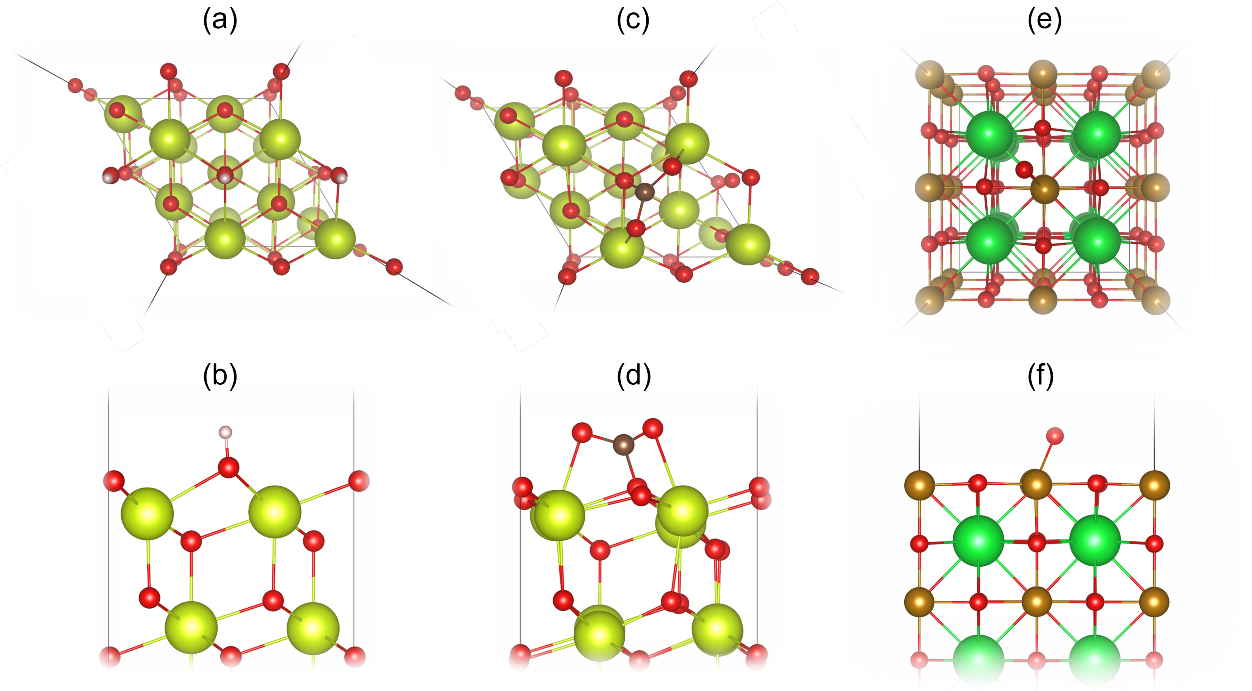


Figure S2: Schematic of the adsorbate state for (a-b) $2\mathrm{OH}^{\bullet}_{\mathrm{O}}$ on $CeO_2$, (c-d) $\mathbf{CO_3}^{\mathbf{x}}_{\mathbf{O}}$ on $CeO_2$ and (e-f) $\mathrm{O}'_{\mathrm{O}}$ on $LaFeO_3$, where (a) and (c) shows the [111] projection; (b), (f) and (d) shows the [010] projection, and (e) shows [100] projection. Yellow, red, white, brown, orange and green spheres represent Ce, O, H, C, Fe and La species, respectively.

Table S1:Derivations of the activation overpotential detailed in Table 1.

| $\mathrm{H_2O}$ reduction | |
|---|---|
| $\mathrm{H_2O_{(g)}} + \mathrm{V_O^{\bullet\bullet}} + \mathrm{O_O^x} \rightleftharpoons 2\mathrm{OH_O^{\bullet}}$<br>$2e\eta_{act} = 2\mu_{\mathrm{OH_O^{\bullet}}} - \mu_{\mathrm{H_2O_{(g)}}} - \mu_{\mathrm{V_O^{\bullet\bullet}}} - \mu_{\mathrm{O_O^x}}$<br>$2e\eta_{act} = \Delta\mu^{\mathrm{o}} + k_{\mathrm{B}}T\ln\left(\frac{c_{\mathrm{OH_O^{\bullet}}}{}^2}{p_{\mathrm{H_2O_{(g)}}} c_{V_O^{\bullet\bullet}} c_{\mathrm{O_O^x}}}\right) - 2e\chi$<br>$2e\eta_{act} = -T\Delta S_{conf} - 2e\Delta\chi$ | $\mathbf{2OH_O^{\bullet} + 2Ce_{Ce}' \rightleftharpoons H_{2(g)} + 2O_O^x + 2Ce_{Ce}^x}$<br>$2e\eta_{act} = \mu_{\mathrm{H_{2(g)}}} + 2\mu_{\mathrm{O_O^x}} + 2\mu_{\mathrm{Ce_{Ce}^x}} - 2\mu_{\mathrm{OH_O^{\bullet}}} - 2\mu_{\mathrm{Ce_{Ce}'}}$<br>$2e\eta_{act} = \Delta\mu^{\mathrm{o}} + k_{\mathrm{B}}T\ln\left(\frac{p_{\mathrm{H_{2(g)}}} c_{\mathrm{O_O^x}}{}^2 c_{\mathrm{Ce_{Ce}^x}}{}^2}{c_{\mathrm{OH_O^{\bullet}}}{}^2 c_{\mathrm{Ce_{Ce}'}}{}^2}\right) + 2e\chi$<br>$2e\eta_{act} = -T\Delta S_{conf} + 2e\Delta\chi$ |
| $\mathrm{CO_2}$ reduction | |
| $\mathrm{CO_{2(g)}} + \mathrm{O_O^x} \rightleftharpoons \mathrm{CO_{3}{}_O^x}$<br>$e\eta_{act} = \mu_{\mathrm{CO_{3}{}_O^x}} - \mu_{\mathrm{CO_{2(g)}}} - \mu_{\mathrm{O_O^x}}$<br>$e\eta_{act} = \Delta\mu^{\mathrm{o}} + k_{\mathrm{B}}T\ln\left(\frac{c_{\mathrm{CO_{3}{}_O^x}}}{p_{\mathrm{CO_{2(g)}}} c_{\mathrm{O_O^x}}}\right)$<br>$2e\eta_{act} = -T\Delta S_{conf}$ | $\mathbf{CO_{3}{}_O^x + 2Ce_{Ce}' + V_O^{\bullet\bullet} \rightleftharpoons CO_{(g)} + 2O_O^x + 2Ce_{Ce}^x}$<br>$2e\eta_{act} = \mu_{\mathrm{CO_{(g)}}} + 2\mu_{\mathrm{O_O^x}} + 2\mu_{\mathrm{Ce_{Ce}^x}} - \mu_{\mathrm{CO_{3}{}_O^x}} - \mu_{\mathrm{V_O^{\bullet\bullet}}} - 2\mu_{\mathrm{Ce_{Ce}'}}$<br>$2e\eta_{act} = \Delta\mu^{\mathrm{o}} + k_{\mathrm{B}}T\ln\left(\frac{p_{\mathrm{CO_{(g)}}} c_{\mathrm{O_O^x}}{}^2 c_{\mathrm{Ce_{Ce}^x}}{}^2}{c_{\mathrm{CO_{3}{}_O^x}} c_{\mathrm{V_O^{\bullet\bullet}}} c_{\mathrm{Ce_{Ce}'}}{}^2}\right)$<br>$2e\eta_{act} = -T\Delta S_{conf}$ |
| $\mathrm{O_2}$ reduction | |
| $\frac{1}{2}\mathrm{O_{2(g)}} + \mathrm{Fe_{Fe}^x} \rightleftharpoons \mathrm{O_{ads}'} + \mathrm{Fe_{Fe}^{\bullet}}$<br>$e\eta_{act} = \mu_{\mathrm{O_{ads}'}} + \mu_{\mathrm{Fe_{Fe}^{\bullet}}} - \frac{1}{2}\mu_{\mathrm{O_{2(g)}}} - \mu_{\mathrm{Fe_{Fe}^x}}$<br>$e\eta_{act} = \Delta\mu^{\mathrm{o}} + k_{\mathrm{B}}T\ln\left(\frac{c_{\mathrm{O_{ads}'}} c_{\mathrm{Fe_{Fe}^{\bullet}}}}{\sqrt{p_{\mathrm{O_{2(g)}}}} c_{\mathrm{Fe_{Fe}^x}}}\right) + e\chi$<br>$e\eta_{act} = T\Delta S_{conf} + e\Delta\chi$ | $\mathbf{O_{ads}' + Fe_{Fe}^x + V_O^{\bullet\bullet}} \rightleftharpoons \mathrm{O_O^x} + \mathbf{Fe_{Fe}^{\bullet}}$<br>$e\eta_{act} = \mu_{\mathrm{O_O^x}} + \mu_{\mathrm{Fe_{Fe}^{\bullet}}} - \mu_{\mathrm{O_{ads}'}} - \mu_{\mathrm{Fe_{Fe}^x}} - \mu_{\mathrm{V_O^{\bullet\bullet}}}$<br>$e\eta_{act} = \Delta\mu^{\mathrm{o}} + k_{\mathrm{B}}T\ln\left(\frac{c_{\mathrm{O_O^x}} c_{\mathrm{Fe_{Fe}^{\bullet}}}}{c_{\mathrm{O_{ads}'}} c_{\mathrm{Fe_{Fe}^x}} c_{\mathrm{V_O^{\bullet\bullet}}}}\right) - e\chi$<br>$e\eta_{act} = T\Delta S_{conf} - e\Delta\chi$ |

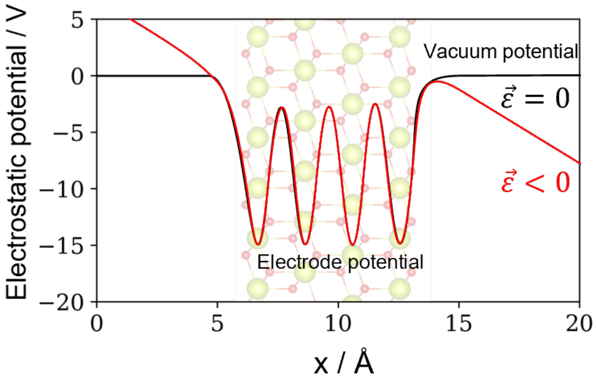


Figure S3: Schematic illustration of the potential with no applied field (black line) and an applied field (red line).

Table S2: Fermi energy, internal energy and electrostatic surface potential of reaction as a function of electric field vector. The penultimate row is the experimentally determined electrostatic surface potential response to an applied overpotential.[1–3]

| | $H_2O$ reduction | $CO_2$ reduction | $O_2$ reduction |
|---|---|---|---|
| $\frac{\partial \Delta E_{\mathrm{F}}(\vec{\mathcal{E}})}{\partial \vec{\mathcal{E}}}$ | -0.34 | -0.26 | 0.16 |
| $\frac{\partial \Delta U_{rxn}(\vec{\mathcal{E}})}{\partial \vec{\mathcal{E}}}$ | -0.46 | -0.14 | 0.09 |
| $\frac{\partial \Delta \chi(\vec{\mathcal{E}})}{\partial \Delta \eta_{act}(\vec{\mathcal{E}})}$ | 0.65 | 0.13 | -0.86 |
| $\frac{\partial \Delta \chi}{\partial \Delta \eta_{act}}$ | 0.75 | 0.03 | -0.47 |
| Error/% | 14 | 23 | 54 |

## Derivation of Eq. 6 and 7

Eq. 5 can be rationalised as the difference between the externally controlled potential ($\mu_{res}$) and the internal energy ($\mu_h$):

$$e\eta_{act} = \mu_{res} - \mu_h \tag{S1}$$

The equilibrium between eq. 1 and 2 gives us the cell voltage ($V = \mu_{res}/e$), thus:

$$\mu_{res} = \frac{1}{2}\mu_{\mathrm{H_2O_{(g)}}} + \mu_{\mathrm{e}^-_{Fuel}} + \frac{1}{2}\mu_{\mathrm{O^{2-}_{Air}}} - \frac{1}{2}\mu_{\mathrm{H_{2(g)}}} - \frac{1}{2}\mu_{\mathrm{O^{2-}_{Fuel}}} - \frac{1}{4}\mu_{\mathrm{O_{2(g)}}} - \mu_{\mathrm{e}^-_{Air}} \tag{S2}$$

where we assume fast oxygen ion diffusion and therefore the gradient in electrostatic and chemical potential in the electrolyte is negligible, as such $\mu_{\mathrm{O^{2-}_{Air}}} \approx \mu_{\mathrm{O^{2-}_{Fuel}}}$, and the internal energy is described by the chemical potential of the gas phase (or open circuit voltage), $\mu_h = \frac{1}{2}\mu_{\mathrm{H_2O_{(g)}}} - \frac{1}{2}\mu_{\mathrm{H_{2(g)}}} - \frac{1}{4}\mu_{\mathrm{O_{2(g)}}}$. As such:

$$\mu_{res} = \mu_h + \mu_{e^-_{Fuel}} - \mu_{e^-_{Air}} \tag{S3}$$

By combining eq. S1 with S3 we find the relationship between the activation overpotential and the electrochemical potential of free electrons at each electrode:

$$e\eta_{act} = \mu_{e^-_{Fuel}} - \mu_{e^-_{Air}} \tag{S4}$$

where the total activation overpotential is the sum of overpotentials at the fuel and air electrodes, $\eta_{act} = \eta_{act,Fuel} + \eta_{act,Air}$.

**Fuel electrode:** When we study the fuel electrode (working electrode) the reaction at the air electrode (reference electrode) is assumed to be facile, $\eta_{act,Air} \approx 0$. We also use the air electrode as the reference potential $\mu_{e^-_{Air}} \approx 0$, thus:

$$\eta_{act,Fuel} = \mu_{e^-_{Fuel}} \tag{S5}$$

which is proportional to the Fermi energy $\eta_{act,Fuel} = \Delta \mathrm{E}_F$.

**Air electrode:** When we study the air electrode (working electrode) the reaction at the fuel electrode (reference electrode) is assumed to be facile, $\eta_{act,Fuel} \approx 0$. We also use the fuel electrode as the reference potential $\mu_{e^-_{Fuel}} \approx 0$, thus:

$$\eta_{act,Air} = -\mu_{e^-_{Air}} \tag{S6}$$

which is proportional to the negative of the Fermi energy $\eta_{act,Air} = -\Delta \mathrm{E}_F$.

# References


(1) Feng, Z. A.; Gabaly, F. E.; Ye, X.; Shen, Z.-x.; Chueh, W. C. Fast vacancy-mediated oxygen ion incorporation across the ceria–gas electrochemical interface. *Nature Communications* **2014**, *5*, 1–9.

(2) Feng, Z. A.; Machala, M. L.; Chueh, W. C. Surface electrochemistry of $CO_2$ reduction and CO oxidation on Sm-doped $CeO_{2-x}$: Coupling between $Ce^{3+}$ and carbonate adsorbates. *Physical Chemistry Chemical Physics* **2015**, *17*, 12273–12281.

(3) Feng, Zhuoluo Albert, Operando X-ray Photoelectron Spectroscopy Investigation of Ceria/Gas Electrochemical Interfaces. *Stanford University* **2015**, 1–141.
